# Discovery and description of a mysterious Asian flying squirrel (Rodentia, Sciuridae, *Biswamoyopterus*) from Mount Gaoligong, southwest China

**DOI:** 10.3897/zookeys.864.33678

**Published:** 2019-07-18

**Authors:** Quan Li, Xue-You Li, Stephen M. Jackson, Fei Li, Ming Jiang, Wei Zhao, Wen-Yu Song, Xue-Long Jiang

**Affiliations:** 1 State Key Laboratory of Genetic Resources and Evolution, Kunming Institute of Zoology, Chinese Academy of Sciences, Kunming, China Kunming Institute of Zoology, Chinese Academy of Sciences Kunming China; 2 Kunming College of Life Science, University of Chinese Academy of Sciences, Kunming, China University of Chinese Academy of Sciences Kunming China; 3 Biosecurity NSW, NSW Department of Primary Industries, Orange, New South Wales 2800, Australia Biosecurity NSW Orange Australia; 4 School of Biological, Earth and Environmental Sciences, University of New South Wales, Sydney, New South Wales 2052, Australia University of New South Wales Sydney Australia; 5 Division of Mammals, National Museum of Natural History, Smithsonian Institution, Washington, DC 20013-7012, United States of America National Museum of Natural History, Smithsonian Institution Washington United States of America; 6 Australian Museum Research Institute, Australian Museum, 1 William St. Sydney, New South Wales 2010, Australia Australian Museum Research Institute Sydney Australia; 7 Kadoorie Conservation China, Kadoorie Farm & Botanic Garden, Lam Kam Road, Tai Po, Hong Kong, China Kadoorie Conservation China, Kadoorie Farm & Botanic Garden Hong Kong China; 8 Baoshan Management Bureau of Gaoligongshan National Nature Reserve, Baoshan, Yunnan, China Baoshan Management Bureau of Gaoligongshan National Nature Reserve Baoshan China

**Keywords:** Biodiversity, conservation, mammal, Pteromyini, systematics, taxonomy, threatened, wildlife, Yunnan

## Abstract

The flying squirrels of the tribe Pteromyini (Family Sciuridae) currently include 15 genera of which the genus *Biswamoyopterus* comprises two recognized species, *B.biswasi* Saha, 1981 and *B.laoensis* Sanamxay et al., 2013. These two species were each described from only one specimen that are separated from each other by 1,250 kilometres in southern Asia, where they occur in northeast India and central Lao PDR respectively. In 2017 and 2018, two specimens of *Biswamoyopterus* were discovered from Mount Gaoligong, west Yunnan province, southwest China (between the type locality of the two recognized species). This study aimed to evaluate the taxonomic status of these two newly acquired specimens of *Biswamoyopterus* by comparing their morphology with the two described species of the genus. The results of this study showed that the specimens from Yunnan province (China) differed from both *B.laoensis* and *B.biswasi* in both pelage colour and craniology, and should be recognised as a distinct species, *B.gaoligongensis***sp. nov.**, which is formally described here. This study contributes to the understanding of the flying squirrels of southern Asia and identifies an additional species that appears to be endemic to southwest China; however, more research is required to provide details of its ecology, distribution, and conservation status.

## Introduction

The flying squirrels of the tribe Pteromyini (Family Sciuridae) currently comprise 52 species of recent mammals that are placed in 15 genera. A number of fossil species have also been described and includes in several of the genera containing extant species as well as 13 additional extinct genera (Jackson and Thorington 2012; Jackson and Schouten 2012; Koprowski et al. 2016). The genus *Biswamoyopterus* Saha, 1981 is the most recently described in the tribe and initially only included *Biswamoyopterusbiswasi* Saha, 1981 based on a single specimen collected in Namdapha National Park, northeast India (Saha 1981). *Biswamoyopterusbiswasi* was placed in its own genus by Saha (1981) as it was considered to exhibit a unique combination of characters that distinguish it from other genera including: 1) large body size, cylindrical tail, and well-developed uropatagium (tail membrane or interfemoral membrane) similar to *Petaurista*, *Aeretes* and *Aeromys*; 2) the presence of ear tufts similar to *Belomys* and *Trogopterus*; and 3) cuspidate brachyodont dentition similar to *Hylopetes* and *Aeromys*. In addition to these characters, *Biswamoyopterus* was recognised to have pale-yellow incisors similar to *Aeromys* and *Eupetaurus* (Corbet and Hill 1992). In reference to these characters, Sanamxay et al. (2013) described a second species of *Biswamoyopterus* (*B.laoensis*) based on a single specimen collected from central Lao PDR. So far, all knowledge of *Biswamoyopterus* comes from the morphological description of these two holotypes. As a result, the International Union for Conservation of Nature (IUCN) listed *Biswamoyopterusbiswasi* as critically endangered due to hunting and habitat loss from logging (Molur 2016) and *Biswamoyopteruslaoensis* as data deficient (Kennerley 2017).

**Figure 1.**
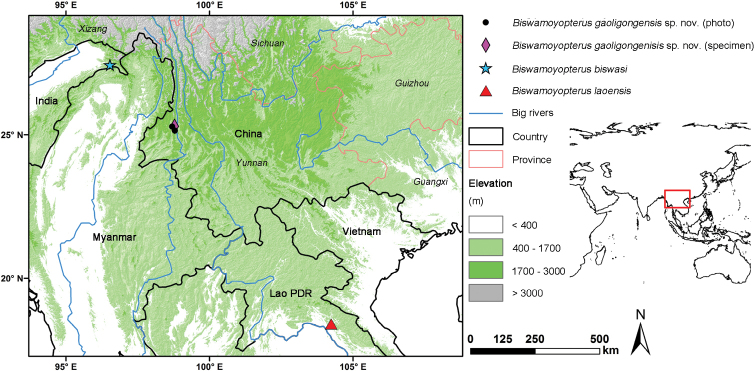
Known localities of three species of *Biswamoyopterus*.

There is a gap of 1,250 km between the type localities of the two described *Biswamoyopterus* species (Sanamxay et al. 2013). Western Yunnan, southwest China occurs between the two type localities of *Biswamoyopterus* (Fig. 1). In 2017 and 2018, two specimens of *Biswamoyopterus* sp. were collected in Mount Gaoligong (the watershed of the Irrawaddy River and the Nu River [Salween River]), west Yunnan (Fig. 1) that appeared to have different pelage and cranial characters from the two described species of *Biswamoyopterus*. Therefore, the aim of this study was to: 1) undertake a detailed comparison of the specimens collected in Yunnan province, China with the two described species; and 2) if these Yunnan specimens proved to be distinct, formally describe and name a new species of *Biswamoyopterus*.

## Materials and methods

### Ethics statement

Animals used for this study were approved by the Animal Ethics Committee of the Kunming Institute of Zoology, Chinese Academy of Sciences (approval ID: SMKX2018021).

### Repositories

**ZSI** Zoological Collection of the Zoological Survey of India, Kolkata [Calcutta], India.

**NUoL** Zoological collection of the Faculty of Environmental Sciences, National University of Laos, Vientiane, Lao PDR.

**KIZ** Kunming Natural History Museum of Zoology, Kunming Institute of Zoology, Chinese Academy of Sciences, Kunming, China.

### Specimens examined

**Holotype**

CHINA • 1♂, holotype of *Biswamoyopterusgaoligongensis* sp. nov., skin and skull available; Yunnan province, Baoshan city, Longyang county, Lujiang township, Baihualin village; 25.298N, 98.785E; 2040 m a.s.l.; Jan. 2017; Quan Li leg.; Broad-leaved evergreen forests; KIZ 034924 (field No. bs1628).

INDIA • 1♂, holotype of *Biswamoyopterusbiswasi*, skin and skull available; Tirap District, Namdapha, 26 km east of Miao, Deban; ca. 350 m a.s.l.; Apr. 1981; Shyamrup Biswas leg.; collected from a tall Nahar tree (*Mesuaferrea*) at 20:15 pm; ZSI 20705.

LAO PDR • 1♀, holotype of *Biswamoyopteruslaoensis*, skin and skull available; Bolikhamxai Province, Pak Kading District, Ban (village) Thongnami, Thongnami market (Purchased from the market by the collectors. The collectors speculated that the original collection site might be Nam Kading National Biodiversity Conservation Areas or Khammouan Limestone National Biodiversity Conservation Areas (NBCA), which is about 5 km Northeast of Thongnami and Khammouan Limestone NBCA, and about 25 km Southeast of Ban village); 18.172N, 104.24E; Sep. 2012; Daosavanh Sanamxay, Sysouphanh Xayavong, and Vilakhan Xayaphet leg.; NUoL FES.MM.12.163.

**Paratype**

CHINA • 1 sex unknown, paratype of *Biswamoyopterusgaoligongensis* sp. nov., skin of head and skull available; same locality as for KIZ 034924; Dec. 2018; a native of the area leg.; KIZ 035622 (field No. 201812001).

### Morphological techniques

**Figure 2.**
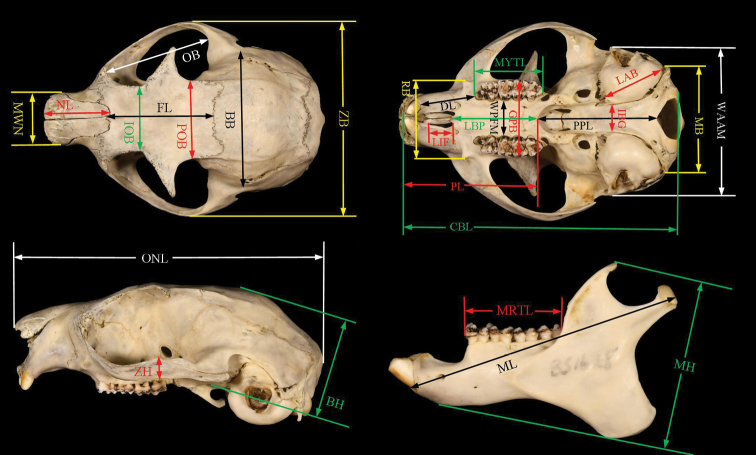
Twenty-eight craniodental measurements taken for this study. See text for definitions. The different coloured arrows have no special meaning, they make it easier to see the starting and ending points of different measurements. Photo credit: Sanamxay et al. (2013).

The external and craniodental measurements of type specimen of *Biswamoyopterusbiswasi* and *Biswamoyopteruslaoensis* were employed from the literature (Saha 1981; Sanamxay et al. 2013). External measurements of *Biswamoyopterus* sp. nov. were copied from the label tied on the specimen, included body mass, head and body length, tail length, hind feet length, and ear length. Craniodental measurements of *Biswamoyopterus* sp. nov. were taken with digital caliper to the nearest 0.01 mm; the mensural points follow Saha (1981) and Sanamxay et al. (2013) to facilitate the subsequent comparison (Fig. 2). A total of 28 craniodental measurements were used, including:

**BB** Breadth of braincase,

**BH** Braincase height,

**CBL** Condylobasal length,

**DL** Diastema length,

**FL** Frontal length,

**GPB** Greatest palatal breadth,

**IBG** Inter bullae gap,

**IOB** Interorbital breadth,

**LAB** Length of auditory bulla,

**LBP** Length of bony palate,

**LIF** Length of the incisive foramina,

**MB** Mastoid breadth,

**MH** Mandible height,

**ML** Mandible length,

**MRTL** Mandibular tooth row length,

**MWN** Maximum width of nasals,

**MYTL** Maxillary tooth row length,

**NL** Nasal length,

**OB** Orbit breadth,

**ONL** Occipitonasal length,

**PL** Palate length,

**POB** Postorbital breadth,

**PPL** Postpalatal length,

**RB** Rostrum breadth,

**WAAM** Width of auditory bullae across the external auditory meati,

**WPFM** Width of the bony palate at the first upper molar,

**ZB** Zygomatic breadth,

**ZH** Zygomatic height.

**P** Premolars,

**M** Molars;

Superscript (P^X^, M^X^) upper premolars and upper molars, and

Subscript (P_X_, M_X_) lower premolars and lower molars.

**Figure 3.**
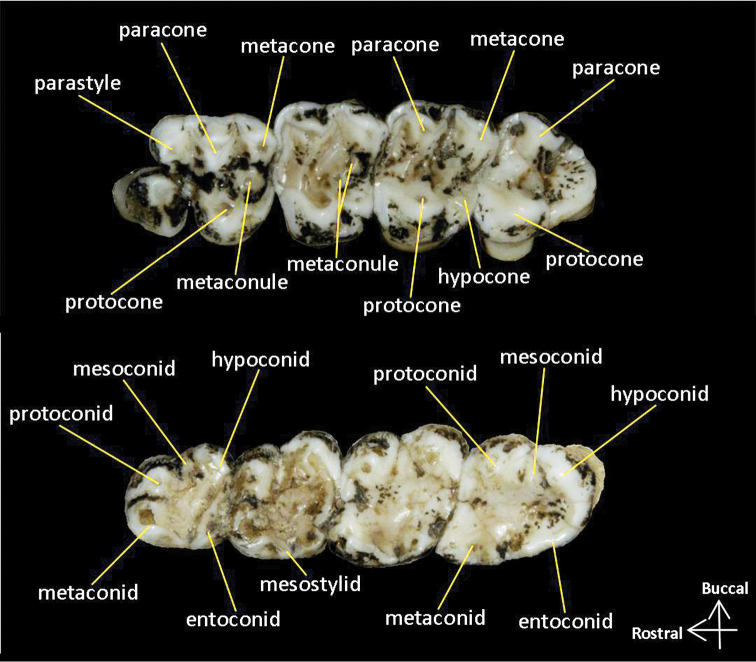
Nomenclature of cheek teeth of *Biswamoyopterus*. Maxillary tooth row (top), Mandibular tooth row (bottom).

The nomenclature of cheek teeth structures followed Tong (2007) and Thorington et al. (1996) (Fig. 3).

Pelage colour comparisons were made among all four available specimens. Skull and teeth were studied using a stereo binocular microscope. As only four skull specimens were available, statistical analysis was not possible.

## Taxonomy

### Class Mammalia Linnaeus, 1758

#### Order Rodentia Bowdich, 1821

##### Family Sciuridae Fischer, 1817

###### Subfamily Sciurinae Fischer, 1817

####### Tribe Pteromyini Brandt, 1855

######## Genus *Biswamoyopterus* Saha, 1981

######### Biswamoyopterus gaoligongensis sp. nov.

Taxon classificationAnimaliaRodentiaSciuridae

7ef40b46-0c5f-401b-aa93-fdb109e0d376

http://zoobank.org/21C9D58C-EDC9-4016-8148-3F81DB51D9D3

########## Common name.

Mount Gaoligong Flying Squirrel. Chinese common name "高黎贡比氏鼯鼠".

########## Holotype.

Specimen KIZ: 034924 (field number bs1628), an adult male, skull, dried skin, baculum, and remaining body part in alcohol deposited in the Kunming Natural History Museum of Zoology, Kunming Institute of Zoology, Chinese Academy of Science (KIZ).

########## Type locality.

Baihualin village [25.298167N, 98.784683E], Lujiang township, Longyang County, Baoshan City, Yunnan, China. The locality is located on the eastern slope of the southern Mount Gaoligong.

########## Etymology.

The specific name is derived from Mount Gaoligong, the type locality of the new species and –*ensis*, Latin for belonging to.

########## Diagnosis.

*Biswamoyopterusgaoligongensis* sp. nov. can be distinguished from the other two described species of *Biswamoyopterus* by the following combination of traits: 1) The ear tufts at the base of the posterior margins of ears are bicolored, basally white and terminal black. The scrotum is dark brown which strongly contrasts with the yellowish-white abdominal pelage. 2) The muzzle is very short, and the zygomatic arch is distinctly expanding outward, making the outline of the skull short and wide. The outer margin of the nasal bone, the orbital margin of the frontal bone, and the post-orbital margin of the frontal bone are almost parallel to the midline of skull on the dorsal view. The central point of the posterior margin of the palatal bones lies in front of the posterior margin of M^3^. 3) M^1^ and M^2^ are sub-square in outline, and as large as P^4^. The hypoconid of P_4_-M_2_ are very developed, strongly pointed towards posterior buccal side.

########## Description.

**Figure 4.**
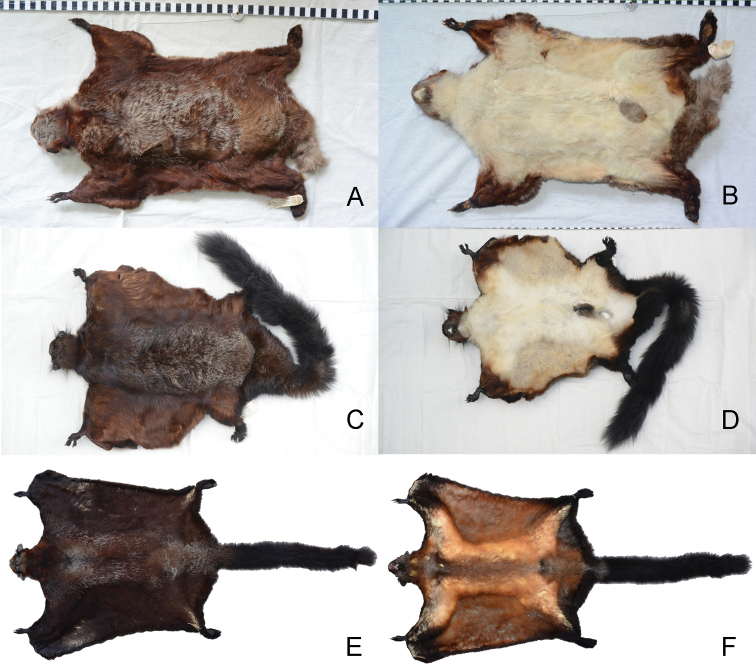
Skins of the three known *Biswamoyopterus* species **A, B** (ZSI 20705, holotype) *Biswamoyopterusbiswasi***C, D** (KIZ 034924, holotype) *Biswamoyopterusgaoligongensis* sp. nov. **E, F** (NUoL FES.MM.12.163, holotype) *Biswamoyopteruslaoensis*. The images **E, F** were derived from Sanamxay et al. (2013).

*Biswamoyopterusgaoligongensis* sp. nov. is a large flying squirrel (head and body length: 440 mm, tail length: 520 mm, and body mass: 1370 g) with a very developed uropatagium that extends approximately one-third of the proximal tail length in fresh specimen (Fig. 4). The back and upper surface of patagium are predominantly reddish brown, while the back between the shoulder and uropatagium is speckled with numerous white-tip furs that are absent from the head, shoulder, plagiopatagium, outer edge of uropatagium, limbs, and tail (Fig. 4). Similar to the shoulder, the head is reddish brown, but showing some yellowish grey in the crown. The ear is naked, with two bunches of long hairs (i.e., ear tufts) at the ear base, the anterior tufts are black, and the posterior tufts are basally white and terminal black. The back of each manus is reddish brown and the digits are black, while the whole pes and digits are black. The tail is cylindrical, the part beyond the uropatagium is black, and the part within the uropatagium is the same colour as the uropatagium. Throat, belly, and ventral surface of patagium are yellowish white. However, the scrotum is dark brown which strongly contrasts with the abdominal pelage.

**Figure 5.**
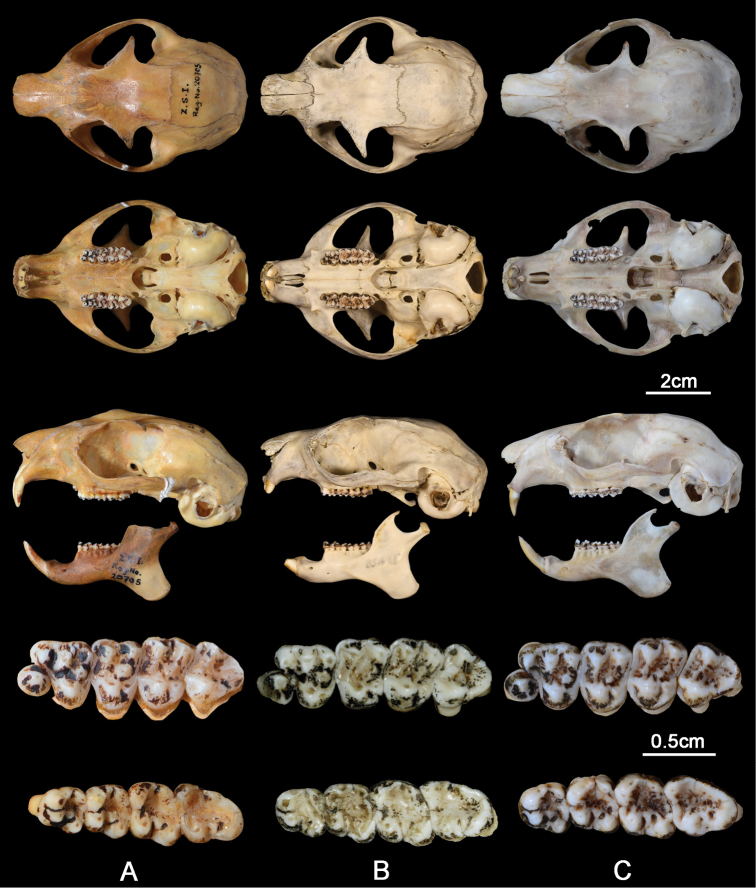
Skulls, left maxillary (above) and left mandibular (below) tooth rows of the three known *Biswamoyopterus* species. **A** (ZSI 20705, holotype) *Biswamoyopterusbiswasi***B** (KIZ 034924, holotype) *Biswamoyopterusgaoligongensis* sp. nov. **C** (NUoL FES.MM.12.163, holotype) *Biswamoyopteruslaoensis*. The images of **C** were derived from Sanamxay et al. (2013).

Skull is large with a short muzzle and an expanded outward zygomatic arch, making the outline of skull short and wide (Fig. 5). The frontal depression is deep and postorbital processes are large and well developed. The outer margin of the nasal bone, the orbital margin of the frontal bone, and the post-orbital margin of the frontal bone are almost parallel to the midline of skull on the dorsal view. The auditory bullae are relatively large, with a honeycomb pattern of complex septae. The interpremaxillary foramen is well opened, which is not common in most flying squirrel genera. The mandible is generally similar to that of other flying squirrels. The coronoid process is less developed, only slightly higher than condylar process when the mandible is placed on a plane.

The anterior surface of incisors is pale yellow. Cheek teeth are strongly cuspidate brachyodont, with slightly pitted enamel.

Maxillary teeth: P^3^ is strong and unicuspid. Parastyle is prominent on P^4^ and dwindle on the following molars in an anterior to posterior gradient. Paracone is prominent on P^4^, M^1^, M^2^, and M^3^. Metacone is prominent on P^4^, M^1^, and M^2^, and indistinct on M^3^. Between protocone and metacone, at the exit of the middle valley of P^4^, M^1^, M^2^, and M^3^, there are two mesostyles form a projecting gutter. Protocone is prominent on P^4^, M^1^, M^2^, and M^3^. Hypocone is small, separated from protocone by a notch, distinct on M^1^ and M^2^, small on P^4^, and absent on M^3^. The anteroloph and posteroloph are indistinct on P^4^ and M^3^; distinct on M^1^ and M^2^, but they do not develop into a ridge as high as the protoloph and metaloph. A protoloph connecting the protocone with the paracone on M^1^, M^2^, and M^3^, and notched on P^4^. A metaloph connecting the protocone with the metacone on M^2^, interrupted by one big or two small metaconules on P^4^ and M^1^, and absent on M^3^.

Mandibular teeth: Four main cusps (protoconid, hypoconid, metaconid, and entoconid) are all distinct on P_4_, M_1_, M_2_, and M_3_. Mesoconid is present on the buccal side of P_4_, M_1_, M_2_, and M_3_, the notch between mesoconid and hypoconid is distinct, seems to be formed by the intense wear and tear. Mesostylid is small and fused with metaconid on P_4_ and M_1_, indistinct on M_2_ and M_3_.

########## Comparison.

**Figure 6.**
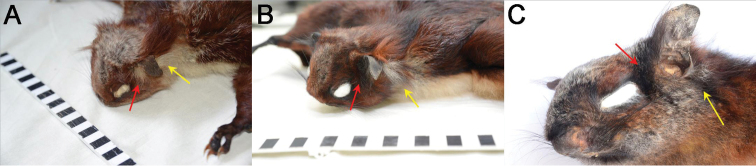
Ear tufts of the three *Biswamoyopterus* species, the red arrow indicates the anterior tufts, and the yellow arrow indicates the posterior tufts **A** (ZSI 20705, holotype) *Biswamoyopterusbiswasi***B** (KIZ 034924, holotype) *Biswamoyopterusgaoligongensis* sp. nov. **C** (NUoL FES.MM.12.163, holotype) *Biswamoyopteruslaoensis*. The image **C** was derived from Sanamxay et al. (2013).

Body size, *B.gaoligongensis* sp. nov. is similar to *B.biswasi* but clearly smaller than *B.laoensis* (Table 1). Pelage colour becomes dark gradually from *B.biswasi* to *B.gaoligongensis* sp. nov. and to *B.laoensis*. The back, *B.biswasi* is morocco-red speckled with white, *B.gaoligongensis* sp. nov. is reddish brown speckled with white, and *B.laoensis* is dark reddish brown speckled with white. The belly, *B.biswasi* is white, *B.gaoligongensis* sp. nov. is yellowish-white, and *B.laoensis* is pale orange. The tail beyond uropatagium, *B.biswasi* is pale smoky grey, with a dark tip, both *B.gaoligongensis* sp. nov. and *B.laoensis* are black (Fig. 4). The ear tufts, *B.biswasi* are white, *B.gaoligongensis*sp. nov. are bicolour (the anterior tufts are black, and the posterior tufts are basally white and terminal black), and *B.laoensis* are black (Fig. 6).

**Table 1.** Body Mass (in grams), external and skull measurements (in mm) of four specimens of genus *Biswamoyopterus*.

Measurements	*B.biswasi* (ZSI 20705)	*B.gaoligongensis* sp. nov. (KIZ 034924)	*B.gaoligongensis* sp. nov. (KIZ 035622)	*B.laoensis* (NUoL FES.MM.12.163)
Body Mass	–	1370.0	–	1800.0
Head and body length	405.0	440.0	–	455.0
Tail length	605.0	520.0	–	620.0
Hind feet length	78.0	75.0	–	74.5
Ear length	46.0	47.0	46.0	52.0
Occipitonasal length (ONL)	72.40	69.75	71.11	74.39
Condylobasal length (CBL)	70.10	66.37	67.73	70.99
Mastoid breadth (MB)	–	30.72	33.50	30.79
Zygomatic breadth (ZB)	47.50	48.41	48.30	47.72
Zygomatic height (ZH)	–	4.61	4.58	4.86
Breadth of braincase (BB)	–	33.86	34.46	32.84
Braincase height (BH)	–	22.90	24.15	22.55
Rostrum breadth (RB)	–	19.61	19.62	17.04
Nasal length (NL)	20.90	19.35	20.70	22.57
Maximum width of nasals (MWN)	–	13.15	12.51	13.37
Interorbital breadth (IOB)	19.00	15.75	16.38	14.06
Postorbital breadth (POB)	–	18.87	20.55	17.19
Length of the incisive foramina (LIF)	6.40	5.65	5.86	5.85
Length of bony palate (LBP)	–	20.08	22.01	23.83
Post palatal length (PPL)	–	28.72	29.68	28.77
Length of auditory bulla (LAB)	15.50	14.68	14.57	17.33
Width of auditory bullae across the external auditory meati (WAAM)	–	35.88	36.76	35.96
Inter bullae gap (IBG)	–	6.52	6.76	5.01
Maxillary tooth row length (MYTL)	15.50	15.92	16.23	16.33
Greatest palatal breadth (GPB)	–	18.26	18.61	19.37
Width of the bony palate at the first upper molar (WPFM)	–	8.58	8.03	8.05
Mandibular tooth row length (MRTL)	–	15.24	15.41	15.33
Mandible length (ML)	–	44.44	46.53	45.36
Mandible height (MH)	–	27.10	27.37	29.78
Palate length (PL)	34.70	32.60	32.87	–
Diastema length (DL)	15.70	13.70	15.03	–
Orbit breadth (OB)	24.60	26.17	26.50	–
Frontal length (FL)	28.60	27.66	30.63	–

**Figure 7.**
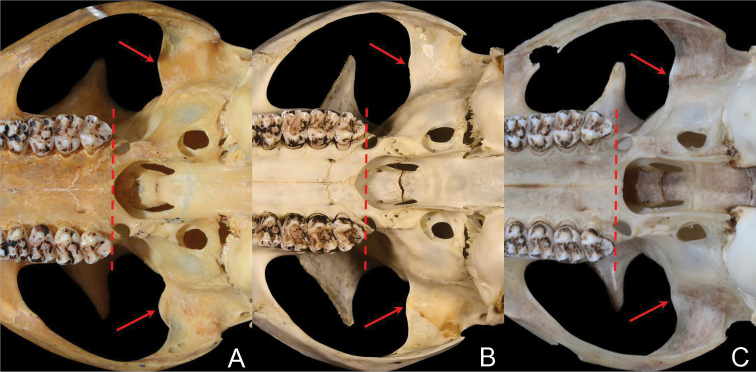
The posterior margin of the palatal bones relative to the posterior margin of M^3^ (dotted line) and shape of the preglenoid process (arrow) of the three *Biswamoyopterus* species **A** (ZSI 20705, holotype) *Biswamoyopterusbiswasi***B** (KIZ 034924, holotype) *Biswamoyopterusgaoligongensis* sp. nov., **C** (NUoL FES.MM.12.163, holotype) *Biswamoyopteruslaoensis*. The image **C** was derived from Sanamxay et al. (2013).

The muzzle of *B.gaoligongensis* sp. nov. is very short, *B.biswasi* is intermediate, and *B.laoensis* is much longer (Fig. 5, Table 1). As a result, the outline of skull of *B.gaoligongensis* sp. nov. is short and wide, *B.biswasi* is relatively short, and *B.laoensis* appears long. On the dorsal view of skull, the outer margin of the nasal bone, the orbital margin of the frontal bone, and the post orbital margin of the frontal bone of *B.gaoligongensis* sp. nov. are almost parallel to the midline of skull, while *B.biswasi* slanted, and *B.laoensis* slanted even more. The postorbital processes of *B.gaoligongensis* sp. nov. and *B.biswasi* are clearly larger than *B.laoensis*. The preglenoid process of *B.gaoligongensis* sp. nov. and *B.laoensis* are almost flat, whereas that of *B.biswasi* obviously protruding forward (Fig. 7). The sutures of frontal and squamosal bone of *B.gaoligongensis* sp. nov. are bulging, while *B.biswasi* and *B.laoensis* are almost flat. The auditory bullae of *B.gaoligongensis* sp. nov. and *B.biswasi* are distinctly smaller than those of *B.laoensis*. The posterior margin of the palatal bones of *B.gaoligongensis* sp. nov. and *B.biswasi* is concave forward, while *B.laoensis* is flat. The central point of the posterior margin of the palatal bones of *B.gaoligongensis* sp. nov. lies in front of the posterior margin of M^3^, *B.biswasi* just meet, and *B.laoensis* lies behind (Fig. 7).

The metacone and hypocone of M^1^ and M^2^ of *B.gaoligongensis* sp. nov. are most developed among three species, followed by *B.laoensis*, again *B.biswasi*. As a result, M^1^ and M^2^ of *B.gaoligongensis* sp. nov. are almost equal to P^4^, while those of *B.laoensis* and *B.biswasi* are smaller than P^4^. In addition, the outline of M^1^ and M^2^ of *B.gaoligongensis* sp. nov. is sub-square, *B.laoensis* is sub-rectangle, and *B.biswasi* is sub-triangular. The hypoconid of *B.gaoligongensis* sp. nov. is strongest among three species, followed by *B.biswasi*, again *B.laoensis* (Fig. 5).

########## Distribution.

Apart from the locality of the holotype, there are two more localities in Yunnan, China, where the *Biswamoyopterusgaoligongensis* sp. nov. was photographed. These include Linjiapu (25.28693N, 98.70102E), 10 km west of the type locality; and Banchang (25.145876N, 98.796026E), 9 km south of the type locality (Fig. 1). Although these three localities cover the east and west slopes of Mount Gaoligong (the watershed of the Irrawaddy River and the Nu River [Salween River]), they are all restricted in a small area of southern Mount Gaoligong.

########## Natural history.

Little is known about the natural history of *Biswamoyopterusgaoligongensis* sp. nov. The holotype was collected from evergreen broad-leaved forest at an altitude of 2,000 meters above sea level. A set of photos taken in Linjiapu showed a *Biswamoyopterusgaoligongensis* sp. nov. resting on the branches of *Daphniphyllum* sp. *Petauristayunanensis*, *P.elegans*, and *Hylopetesalboniger* were also collected in the same habitat where the holotype was collected.

########## Conservation status.

The limited available information suggests that *Biswamoyopterusgaoligongensis* sp. nov. has a relatively low abundance. Because low-altitude forests inhabited by *Biswamoyopterusgaoligongensis* sp. nov. are close to human settlements, they are vulnerable to human activities. The currently known threats are agricultural reclamation and poaching.

######## Key to the three known species of *Biswamoyopterus*

**Table:** 

1	Pale orange belly and marked with numerous, black, discontinuous lines; ear tufts black; long muzzle; large auditory bulla; the posterior edge of the palatal bones is flat	*** Biswamoyopterus laoensis ***
–	Light-coloured belly; ear tufts bicolour or white; short muzzle; smaller auditory bulla; the posterior edge of the palatal bones is concave forward	**2**
2	Parti-coloured tail with a dark tip; ear tufts white; the central point of the posterior margin of the palatal bones just meet the posterior margin of M^3^; the outline of M^1^ and M^2^ is sub-triangular; smaller hypoconid	*** Biswamoyopterus biswasi ***
–	Black tail; ear tufts bicolour; the central point of the posterior margin of the palatal bones lies in front of the posterior margin of M^3^; the outline of M^1^ and M^2^ is sub-square; strong hypoconid	***Biswamoyopterusgaoligongensis* sp. nov.**

## Discussion

This study describes a third species of *Biswamoyopterus* in the middle of the isolated ranges of two previously known species, suggesting that the distribution of *Biswamoyopterus* is much broader than previously known. Although the genetic analysis within *Biswamoyopterus* was not available in this study, the morphological comparison shows that *Biswamoyopterusgaoligongensis* sp. nov. markedly differs from *Biswamoyopterusbiswasi* and *Biswamoyopteruslaoensis* in pelage colour and craniodental traits (Figs 4, 5; Table 2). Within the distribution of *Biswamoyopterus* and adjacent areas (Fig. 1), they occur sympatrically with a number of flying squirrels including *Belomyspearsonii*, *Eupetaurus* sp., *Hylopetesalboniger*, *H.phayrei*, *Petauristaalborufus*, *P.caniceps*, *P.elegans*, *P.petaurista*, *P.philippensis*, *P.yunanensis* and *Trogopterusxanthipes* (Jackson and Thorington 2012; Jackson and Schouten 2012). This high diversity of both genera and species may be the result of the region acted both as refugia and diversification centre since the late Miocene (Lu et al. 2013; Mercer and Roth 2003).

**Table 2.** Comparison of the three species of *Biswamoyopterus*.

Species	* B. biswasi *	*B.gaoligongensis* sp. nov.	* B. laoensis *
Size	Relatively small	Relatively small	Large
Dorsal coloration	Morocco-red speckled with white	Reddish brown speckled with white	Dark reddish brown speckled with whitish-grey
Ventral Coloration	White	Yellowish-white	Pale orange and marked with numerous, black, discontinuous lines
Coloration of tail beyond the uropatagium	Pale smoky grey with a dark tip	Black	Black
Ear tufts	White	The anterior tufts are black, and the posterior tufts are basally white and terminal black	Black
Muzzle	Short	Shorter	Long
Outer margin of the nasal bone, orbital margin of the frontal bone, and post-orbital margin of the frontal bone vs. midline of the skull	Inclined	Almost parallel	More inclined
Postorbital processes	Large	Large	Relatively small
Preglenoid process	Forward protruding	Almost flat	Almost flat
Sutures of frontal and squamosal bone	Almost flat	Bulge	Almost flat
Auditory bulla	Relatively small	Relatively small	Large
Posterior margin of the palatal bones	Concave forward, the central point just meets the posterior margin of M^3^	Concave forward, the central point lies in front of the posterior margin of M^3^	Flat, the central point lies behind the posterior margin of M^3^
M^1^ and M^2^	Feeble metacone and hypocone, outline of M^1^ and M^2^ is sub-triangular	Most developed metacone and hypocone, outline of M^1^ and M^2^ is sub-square	Second developed metacone and hypocone, outline of M^1^ and M^2^ is sub-rectangle
M_1_ and M_2_	Second developed hypoconid	Most developed hypoconid	Feeble hypoconid

## Supplementary Material

XML Treatment for Biswamoyopterus gaoligongensis
